# Multidisciplinary cognitive performance optimization for mission-critical decision makers: delivering “the whole pizza”

**DOI:** 10.3389/fneur.2025.1636406

**Published:** 2025-12-10

**Authors:** David L. Brody

**Affiliations:** Department of Neurology, Uniformed Services University of the Health Sciences (USUHS) and Walter Reed National Military Medical Center, Bethesda, MD, United States

**Keywords:** cognitive performance, sleep, stimulants, lifestyle, caffeine

## Abstract

Despite the promise of artificial intelligence, there remains no substitute for human analytical acumen and decision-making. Human cognitive performance is not fixed; it can often be substantially improved through specific interventions. The field of cognitive performance optimization is multidisciplinary, often involving contributions from physicians, psychologists, athletic trainers, therapists, executive coaches, and many other professionals. Key domains include lifestyle factors, treatment of potentially cognitively impairing disorders, careful use of stimulants, and additional personalized interventions. Modifiable lifestyle factors such as sleep, physical exercise, toxic substances, stress management, and diet play a crucial role. Treatable cognitively impairing disorders include sleep apnea, restless leg syndrome, major depressive disorder, persistent depressive disorder, seasonal affective disorder, anxiety disorders, post-traumatic stress disorder (PTSD), migraine with or without neurological aura, vitamin B12 deficiency, hypothyroidism, and obesity. Stimulants, including both prescription stimulants like methylphenidate and non-prescription stimulants like caffeine, can improve fatigue, sustained attention and processing speed with manageable risks when used carefully. Personalized interventions include correction of vision and hearing deficits, cognitive rehabilitation under professional therapist guidance, computer-based cognitive skills training, cognitive performance coaching, and non-invasive brain stimulation. Taken together, addressing these domains in a multidisciplinary fashion can result in meaningful benefits in terms of cognitive performance. However, cognitive performance optimization requires substantial time and effort on the part of the providers and participants as there are rarely any “easy fixes.” Importantly, cognitive performance optimization is an evolving science with great promise for future advanced interventions.

## Introduction

Key personnel in the US Military, the Federal Government, and many other organizations are required to perform challenging cognitive tasks for which the potential consequences of errors can be high. Thus, the topic of cognitive performance optimization is becoming increasingly recognized as critical for national security. In 2022, the Department of Defense launched the Warfighter Brain Health Initiative with the goal to “bring together the operational and medical communities in a more unified approach toward optimizing service member brain health and countering traumatic brain injuries.” The focus for the Warfighter Brain Health Initiative includes “optimizing cognitive and physical performance” (https://www.health.mil/Military-Health-Topics/Warfighter-Brain-Health, accessed 13 NOV 2025).

Despite the promise of artificial intelligence, there is still, as yet, no substitute for human analytical acumen and decision-making. Furthermore, it is now widely recognized that human cognitive performance is not fixed, nor does it inevitably decline or vary with age. Instead, some aspects of cognitive performance can be actively optimized through a multidisciplinary combination of pharmacological and non-pharmacological methods. Future interventions may include surgical approaches such as brain computer interfaces and brain stimulators designed for cognitive performance enhancement.

The term “cognitive performance” can be defined in many ways. In this article, it is defined in the context of roles and responsibility of mission-critical decision makers from the US Federal Government who have sought advice from the author, a neurologist, at the National Intrepid Center of Excellence at Walter Reed National Military Medical Center. Some cognitive performance optimization interventions have been provided in the context of recovery from concussion/”mild” TBI (1), while others have been provided independent of any specific brain injuries or exposures. It is likely that many of the approaches will be similarly applicable to decision-makers in other domains, though the range of definitions is likely to be even broader in the general population. The assessment and treatment approach presented emphasizes domains where there are indications that cognitive performance can often be substantially optimized using combined pharmacological and non-pharmacological approaches, e.g., for attention deficit, cognitive endurance, and social cognition. Other cognitive performance domains such as long-term memory, creativity, and mathematical intuition currently have fewer effective interventions, which underscores the need for continued research.

The question arises about who should be in charge of managing cognitive performance optimization. Psychologists, athletic trainers, therapists, executive coaches, and many professionals contribute. However, when medical diagnoses and pharmacological interventions are key components, physician leadership is necessary. The purpose of this article is therefore to provide the perspective of a physician with specialized experience in cognitive neurology.

## “The whole pizza”

**Figure 1:**
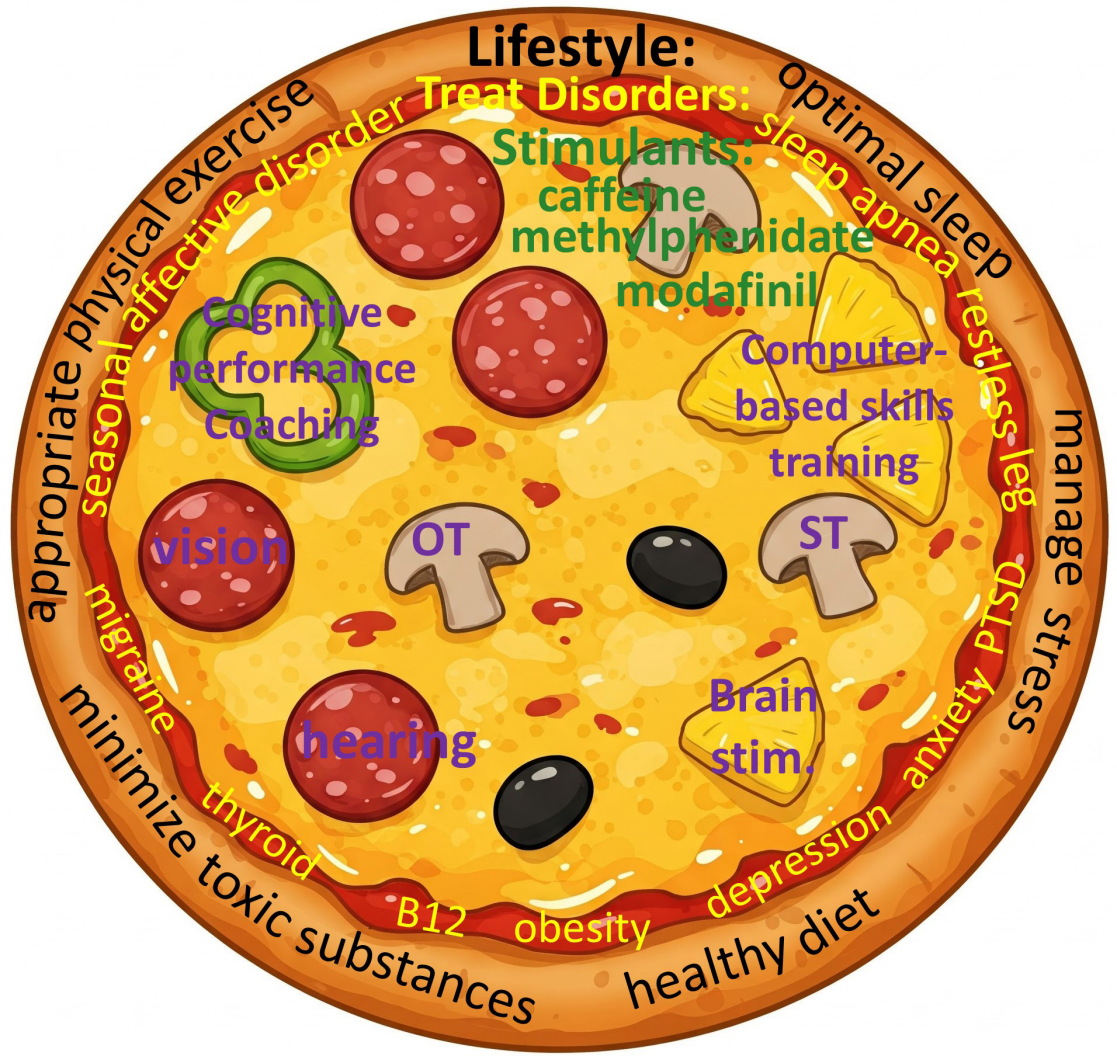
“The whole pizza,” for multidisciplinary cognitive performance optimization. In this analogy, the crust represents lifestyle factors, the sauce represents treatment of cognitively impairing disorders, the cheese represents optimal stimulant management, and the toppings represent personalized specific interventions.

Cognitive performance optimization can be likened to delivering a pizza. All the ingredients go together to make a delicious dish. By analogy, there is no one single intervention that works by itself to optimize cognitive performance, but all the core components, plus a wide variety of extras added on top, combine to produce the final result (Figure 1).

So, what are the ingredients?

**Figure 2:**
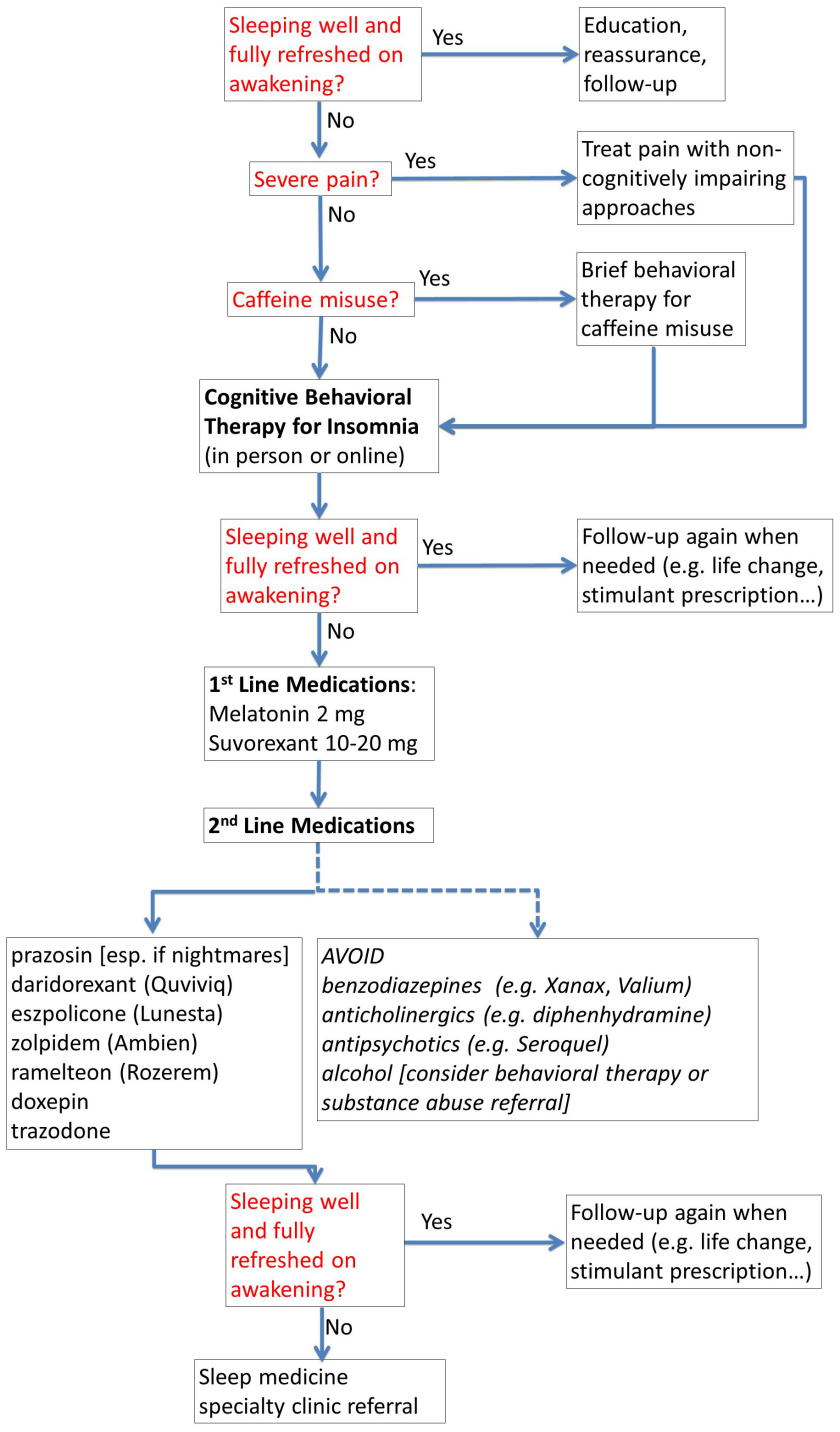
Treatment of insomnia for cognitive performance optimization. The flow chart provides a structured approach to management for non-specialists, including when to refer to a specialty sleep clinic.

**Figure 3:**
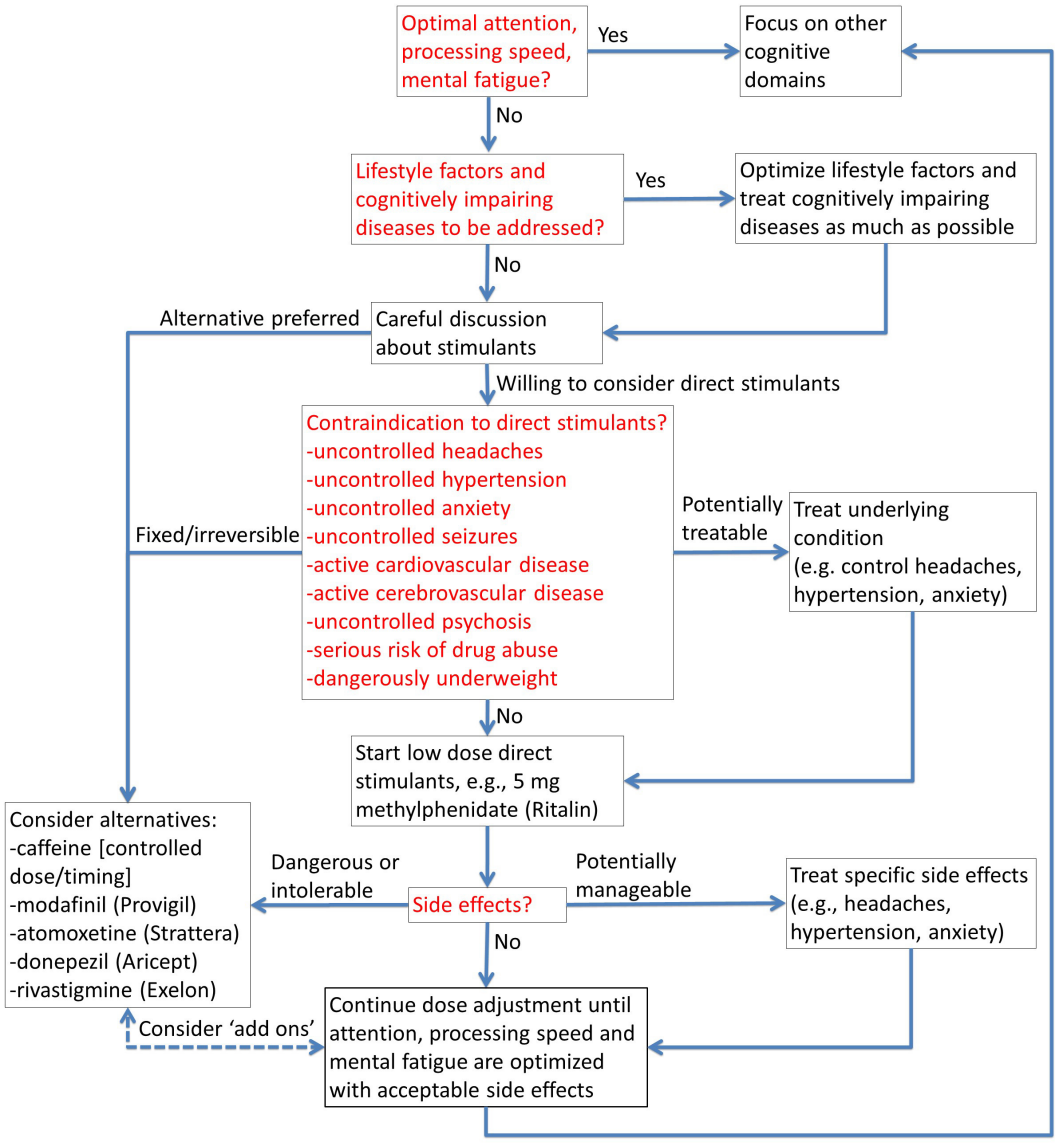
Stimulants for cognitive performance optimization. The flow chart provides a structured approach to safe and appropriate use of prescription stimulants, and alternatives when contraindicated.

1) Lifestyle factors such as good quality sleep, appropriate physical exercise, minimizing toxic substances, stress management, and a healthy diet form the foundation of cognitive performance optimization (perhaps analogous to the crust).
  - Sleep. Truly optimal sleep, in our experience, has the largest overall return on investment in terms of improved cognitive performance. Optimizing both quantity and quality of sleep is often the very first priority. Our current view is that there is no “one size fits all” approach to sleep. Most people have been told that they will perform optimally with 7–8 h of good quality sleep. However, recent evidence indicates that even more sleep, up to 10 h, improves performance further (2–4). Likewise, some people function optimally sleeping mainly at night, and some do best with an added afternoon nap. However, getting optimal sleep is not easy. For those who do not have a specific sleep disorder (e.g., sleep apnea, restless leg), treatment for insomnia is the mainstay. Our approach to insomnia is illustrated in Figure 2. Importantly, cognitive behavioral therapy for insomnia is now available from a smart phone app called “CBT-I Coach” (https://mobile.va.gov/app/cbt-i-coach). It can be difficult to find trained in-person or telemedicine providers available in a timely fashion, so online treatment represents a good option that can be initiated right away. Mission critical decision leaders who have to travel often require additional treatments including morning bright light therapy and sleep banking in advance (3).
  - Physical exercise: Scientific evidence from randomized controlled trials continues to grow in support of the benefits of physical exercise for cognitive performance (5, 6). We often provide a written prescription for “30–60 min of moderately intense exercise every day, whether you feel like it or not.” The specific form of exercise (e.g., cardiovascular, strength training, resistance bands) seems to be less important. Likewise, several 5–10 min “exercise snacks” throughout the day can be a pragmatic alternative if 30–60 min in one session is not feasible. Even a few minutes a day of light exercise like walking is better than being completely sedentary.
  - Alcohol use: Evidence also continues to grow that even relatively small quantities of alcohol can impair cognitive performance (7). For optimal cognitive performance, most people should typically not drink more than 1 alcoholic drink in a 24-h period. More alcohol than that can cause residual cognitive impairment and reduce sleep quality. We typically use motivational interviewing to bring up this topic with patients interested in cognitive performance optimization. Patients often come to the conclusion on their own that the optimal amount of alcohol is none. Intriguingly, Glucagon-like peptide-1 (GLP-1) agonists prescribed for weight loss (see section below on treatment of obstructive sleep apnea) also can substantially reduce alcohol use (8).
  - Stress management: Stress management is important, but requires a careful balance of factors given the complex relationship between arousal and cognitive performance. Under some circumstances, moderate “stress” (aka arousal) improves cognitive performance, whereas extreme stress can often be detrimental (9). While recognized as an over-simplification, it can be helpful to draw the inverted *U*-shaped version of the Yerkes-Dodson curve (stress on the *X*-axis and performance on the *Y*-axis) and used motivational interviewing to inquire about where the patient finds themselves during times of suboptimal cognitive performance. Some are under-aroused (bored), some are overstressed, and some have both issues under different circumstances. In our experience, meditation is among the most powerful tools for modulating arousal, but it is not easy. We often recommend the book *The Wise Heart* (10) as a place to start, with the expectation that it may take 6–12 months to see benefits. There is evidence for the benefits of yoga (11) and breath training (12) in this domain as well.
  - Diet, nutrition, and supplements: A healthy diet is associated with improved cognitive performance, though effects are relatively modest in comparison with other interventions (13). Healthy diet options include traditional Mediterranean diets; diets rich in fatty fish; plant-based diets involving fruits, vegetables, nuts and whole grains; and ketogenic diets. All have been associated with improved cognitive performance. However, there does not seem to be a single “best” diet for cognitive function; there is room for personal preference. Dietary supplements considered potentially beneficial can include creatine monohydrate (up to 25 g/day), omega-3 fatty acids, beta-alanine (up to 6 g/day), and L-tyrosine (up to 300 mg/kg/day) (14). Again, effects are relatively modest in isolation. Caffeine can be considered a dietary supplement as well, but we consider it a stimulant (see below).
2) Treatment of cognitively impairing disorders is a requirement when these conditions are present (perhaps analogous to the sauce). In our experience, even generally healthy adults often have one or more treatable medical conditions that affect optimal cognitive performance. Relatively common, underrecognized, and treatable conditions include sleep apnea, restless leg syndrome, major depressive disorder, persistent depressive disorder, seasonal affective disorder, anxiety disorders, post-traumatic stress disorder (PTSD), migraine with or without neurological aura, vitamin B12 deficiency, hypothyroidism, and obesity. We typically perform the following evaluations in most patients seeking cognitive performance optimization:
  - Sleep studies: Polysomnography, either in the lab or at home, should be performed in nearly everyone. In our experience, obstructive sleep apnea is a very common cause of cognitive performance impairment. Airway management (CPAP, BiPAP) can be helpful, but optimal treatment often requires substantial weight loss. Importantly, the GLP-1 agonist tirzepatide has been shown to be very effective for weight loss in the context of sleep apnea (15), and it is now FDA approved for this indication.
  - Clinical evaluations for neurological and psychiatric conditions: Systematic structured clinical evaluations for restless leg syndrome, depression symptoms, seasonal affective disorder, anxiety symptoms, PSTD, and migraine should be performed routinely. Optimal treatment of these conditions can be complex and may require multidisciplinary care.
  - Laboratory testing: Blood tests for vitamin B12 and TSH should be obtained, plus comprehensive metabolic panel, complete blood count, and autoimmune antibody testing if not done by other providers.
  - Measures of obesity: Measurements of obesity include waist circumference, waist-to-hip ratio, and body mass index (BMI). Obesity is now recognized as an independent contributor to suboptimal cognitive performance even in the absence of other co-occurring conditions (16). BMI alone is not an appropriate measure of obesity for muscular individuals like many military service members, so waist-to-hip ratio is preferred; intervention is indicated for waist-to-hip ratios >1 in men and >0.8 in women.
  - Medication optimization: In addition, we work carefully with other health care providers to assess whether ongoing treatment for other conditions can be optimized to reduce cognitive side effects of medications. Common examples:
    ○ Sedating antihistamines can be replaced by non-sedating antihistamines.
    ○ Benzodiazepine doses can be reduced and supplemented with less-sedating alternatives for anxiety disorders. Physical exercise also has anxiolytic effects.
    ○ Anticholinergic over the counter medications for insomnia like diphenhydramine can be replaced with less cognitively impairing medications like melatonin and suvorexant, while initiating cognitive behavioral therapy for insomnia.
    ○ Cognitively impairing migraine prophylactic medications like topiramate and valproic acid can be replaced with botulinum toxin injections and calcitonin gene related peptide (CGRP)-pathway agents (injectable monoclonal antibodies and oral medications), which do not typically cause cognitive side effects.

3) Stimulants, including both prescription stimulants like methylphenidate and non-prescription stimulants like caffeine, can be extremely effective in domains like fatigue, sustained attention and processing speed (17–19). Stimulants, when not contraindicated, should be considered carefully in nearly every case. (There is usually some kind of cheese on most pizzas.) Our approach to stimulants is illustrated in Figure 3.
- Interactions between stimulants and sleep: Importantly, stimulant use and sleep optimization have to considered together. We typically recommend no caffeine past 8 h before bedtime and no short-acting methylphenidate past 6 h before bedtime. We recommend long acting stimulants only after sleep is well managed, and with careful consideration of their pharmacokinetics. Individual responses to stimulants vary considerably, and some experimentation with dosing and timing is required. Interestingly, we find that methylphenidate effects on sleep are more predictable than caffeine effects on sleep. In part, this may be because the quantities of caffeine are not standardized; it is often hard to know how much caffeine is being used in non-FDA-regulated products like coffee, energy drinks and supplements.
- Contraindications: There are many relative contraindications to direct stimulants like methylphenidate, but most of them can be managed. Typically, the net benefits outweigh the risks in otherwise healthy individuals who require cognitive performance optimization for mission critical activities. Modafinil is a more modestly effective but still potentially beneficial alternative for people who cannot take direct stimulants like methylphenidate (17).
- Caffeine: Caffeine is the most widely used performance enhancing stimulant in the world. We recommend carefully measured doses up to 200–400 mg per day and strict limitation to < 8 h before bedtime (14). Some trial-and-error is required, since the actual dose of caffeine in many products may not be disclosed, or may differ from what is written on the packaging.
- Mood enhancing effects: Stimulants can also have mood enhancing effects, such that subjective impressions of cognitive benefit may be even greater than objectively measured effects (18, 19).
- Caveats: Stimulants are not a panacea; evidence for benefit in cognitive domains other than attention, concentration, processing speed, and mental fatigue are relatively limited. That being said, attention, concentration, processing speed and mental endurance are foundational building blocks upon which performance in other cognitive domains is built.
- Long-term considerations: It is still not known whether stimulants further improve cognitive performance in people who have truly optimized sleep. Anecdotally, some patients have been able to stop stimulant use without any cognitive regression after 6–12 months of fully satiating sleep. Others may continue to take stimulants at stable doses for decades as long as they take at least 1 day off each week and 1 week off each year.
4) Personalized, specific interventions can be useful, but not every intervention is appropriate for everyone (perhaps analogous to toppings).

- Vision and hearing: Correction of vision and hearing deficits can reduce cognitive fatigue in some individuals.
- Cognitive rehabilitation: Cognitive rehabilitation under professional guidance, often by speech therapists, occupational therapists, psychologists or others, can be helpful in some cases. However, these professionals often have greater expertise in guiding recovery from brain insults like traumatic brain injury and stroke, rather than in truly optimizing performance in people who do not necessarily have a specific brain disease.
- Computer-based training: Computer-based cognitive skills training can also be considered (20). This type of training is intensive; 5 h per week for 8–13 weeks were the doses that demonstrated benefit in recent randomized controlled trials of one successful intervention (21). It is important to recognize that many of the training methods produce strong gains in the specific domains being trained, but do not result in generalized cognitive performance improvements or skills relevant to real-world performance (22).
- Cognitive performance coaching: Cognitive performance coaching is a growing field, based on expertise from athletic trainers, executive coaches, and others. This is not always available, and results have been variable. In our experience, social cognition and emotional intelligence are domains that are especially amenable to optimization under the guidance of an appropriately trained coach. In addition, positive self-talk and visualization are methods translated from the sports performance world that can be used to attempt to optimize arousal and self-efficacy in the domain of cognitive performance.
- Brain stimulation: Brain stimulation using relatively safe methods like transcranial direct current stimulation and transcranial alternating current stimulation can be considered. There is relatively limited evidence or experience with these domains, but some are promising (23). Again, in our experience, results have been variable.

Some of these ingredients will be discussed in the context of two brief illustrative examples. Please note that these do not represent specific individuals, but rather are amalgams designed to illustrate key features of common scenarios. More detailed descriptions of the examples are provided in the Supplemental material.

## Example 1: “military intelligence”

A 37-year-old Air Force cryptologist asks for help for problems with concentration. She recounts that she performs intense cognitively demanding work 12 h per day, from 04:30 to 16:30, 3–4 days per week. She notes that her concentration is good at the beginning of her shifts but after about 8 h, it begins to decrement. By the end of her 12-h shifts, she consistently needs to have a co-worker check her work because she is afraid that she may have forgotten or overlooked critical details. She is worried that that an error on her part may cause the US Military to respond late or sub-optimally to an adversary's cyber-domain activities. She drinks caffeinated energy drinks throughout the day. She reports eye strain blurry vision. She has occasional migraine headaches. Her migraines can be both triggered by cognitive overexertion, and also result in subjective cognitive impairment. She reports cogniphobia (24) (“it hurts to think”) and avoids cognitively challenging tasks when she has a migraine. She has a history of significant noise exposure and says her hearing is not as good as it used to be. She sleeps poorly due in part to worry about her work performance. She drinks alcohol on weekends. She exercises on her days off but does not exercise on work days. Her performance is worse in the winter months when she comes in to work in the dark, gets no natural light exposure during the workday, and leaves near sundown. She denies other symptoms of depression or PTSD. After careful evaluations, she is assessed as follows:

- “Relative” attention deficit, meaning that although she may not have a diagnosis of attention deficit disorder as defined in a medical context (i.e. she does not have a “disease”), her capacity for sustained attention is not adequate to meet the requirements of her situation.
- Insomnia secondary to situational anxiety and caffeine misuse.
- Possible alcohol misuse.
- Suboptimal physical exercise.
- Mild visual acuity deficits and mild sensorineural hearing loss.
- Episodic migraine without aura.
- Possible seasonal affective disorder.

After multiple clinic visits and therapeutic trials over 6 months (see supplement for details), her cognitive optimization plan includes the following elements:

- Motivational interviewing with regard to alcohol use, caffeine use, and exercise.
- Cognitive behavioral therapy for insomnia (CBT-I) (25).
- Sustained release melatonin 2 mg each night for insomnia.
- Full spectrum bright light 30–45 min each morning as used to treat seasonal affective disorder (26).
- Prescription of an oral triptan for episodic migraine.
- Titration of methylphenidate to 15 mg each day at 04:30 and 11:00, at most 5 days per week, with no methylphenidate on weekends or holidays.
- Prescription eye glasses and low gain hearing aids.

No single element of her optimization plan is sufficient in isolation, but taken together, they dramatically improve her real-world cognitive performance.

## Example 2: “senior leader”

A 59-year-old senior officer at the Pentagon presents with questions about whether he has “early onset Alzheimer's.” His main concerns are that he has progressively worsening trouble remembering peoples' names, keeping track of details, and organizing his priorities at work. He reports that he has recently been promoted to a position requiring much greater responsibility than he has had in the past, including overseas travel as well as managing a large and fractious staff. His past medical history is notable for hypertension, hypercholesterolemia, and episodic migraine with typical visual aura and difficulty concentrating even after the headaches have resolved. He has a waist-to-hip ratio of 1.2 (meeting criteria for obesity), a BMI of 31, and a 17-inch neck. His wife reports that he snores. He drinks 1–3 alcoholic drinks per night. His brain MRI shows mild generalized atrophy and five small foci of elevated T2/FLAIR signal in the deep white matter.

After careful evaluations, he is assessed as follows:

- Mild cognitive impairment, especially in the domains of executive function and emotional intelligence.
- Obstructive sleep apnea.
- Obesity.
- Episodic migraine with cognitive impairment during postdrome.
- Early cerebrovascular disease.

After multiple clinic visits and therapeutic trials (see supplement for details), his cognitive optimization plan includes the following elements:

- Weight loss using the GLP-1 agonist tirzepatide titrated up to 10 mg/week to address sleep apnea and obesity.
- Abstinence from alcohol- facilitated by the GLP-1 agonist
- Botulinum toxin injections for prophylaxis against migraine with cognitive impairment postdrome
- Leadership coaching for executive function and emotional intelligence
- Jet lag management including sleep banking in advance, suvorexant for occasional insomnia, morning full spectrum bright light plus physical exercise on arrival, and modafinil for wakefulness during the daytime until adapted to the time zone.

Again, no single element of his optimization plan is sufficient in isolation. But after 1-year of management, he feels like he's “finally caught up” after ~40 years of sleep deprivation and his cognitive performance is adequate to meet his responsibilities.

## To summarize the “take home messages”

1) Cognitive performance optimization is a multidisciplinary undertaking.
2) Cognitive performance optimization takes time and effort on the part of the patient. There are usually no easy fixes.
3) Cognitive performance optimization requires time and effort on the part of the provider, with multiple visits and evaluations.
4) Cognitive performance optimization is different for different people. There is no “one size fits all” approach.
5) Cognitive performance optimization is an evolving science. Newer approaches like cognitive performance coaching and GLP-1 agonists for weight loss and alcohol use reduction may play an important role, but the extent of their benefits and their long-term associated risks are not yet well understood.
6) There is great promise for future advanced interventions, though more research and clinical experience will be required to sort out what works and what doesn't.

## Data Availability

The original contributions presented in the study are included in the article/Supplementary material, further inquiries can be directed to the corresponding author.
