# Supplementary material for: Multidisciplinary cognitive performance optimization for mission-critical decision makers: delivering “the whole pizza”
Source: Front Neurol. 2025 Dec 10;16:1636406. doi: 10.3389/fneur.2025.1636406 (PMC12728740; doi:10.3389/fneur.2025.1636406)
Supplement: Supplementary file 1 [file Table_1.docx]

SUPPLEMENTAL MATERIALS:

Example 1: “Military Intelligence”

A 37-year-old Air Force cryptologist with a remote history of concussion and tinnitus but no other medical diagnoses asks for help for problems with concentration. She recounts that she performs intense cognitively demanding work 12 hours per day, from 0430 to 1630, 3-4 days per week most weeks. She has been asked to work the opposite shift from 1630 to 0430 in the past, but no longer does so because she found that it was too difficult. She notes that her concentration is good at the beginning of her shifts but after about 8 hours, it begins to decrement. By the end of her 12-hour shifts, she consistently needs to have a co-worker check her work because she is afraid that she may have forgotten or overlooked critical details. She is worried that that an error on her part may cause the US Military to respond late or sub-optimally to an adversary’s cyber-domain activities. She recounts that she is mentally fatigued and has eye strain by the end of each 12-hour shift. About 1-2 days per month she has a 6/10 intensity headache that comes on in the late afternoon. With these headaches, fluorescent lights and computer screens bother her eyes, and it hurts her head to concentrate on difficult tasks. She does not have visual changes or other neurological symptoms during her headaches. She takes ibuprofen, which partially alleviates the headache, but has to leave work early about once per month. She has these concerns all year long, but her energy level, mood, and cognitive function are worse in the darker months of the year when she starts work before sunrise and ends work near sunset. She works in a secure compartmentalized information facility (SCIF) with no windows. She feels that long-term memory is intact, as is her memory for names, faces, driving directions, and practical skills. She denies concerns about her language skills, interpersonal skills, organizational skills, and ‘big picture’ judgement. She denies depression but notes some anxiety, especially about her cognitive performance; she often has trouble falling asleep and awakens before her alarm goes off due to racing thoughts and worries that she may miss something important that day at work. She states that she drinks 2-3 alcoholic drinks 1-2 nights per week when she does not have to go to work the next day and does not use any other drugs. She states that she does about 1 hour of cardiovascular exercise and an additional 1 hour of strength training on each of her 3 days off work each week. She does not exercise on her work days other than walking back and forth to the parking lot of her building. She drinks 3 energy drinks, each with 150-200 mg of caffeine, per work day at around 0330, 0930, and 1300. She drinks 1-2 energy drinks or cups of coffee in the morning on non-work days. She states that she gets about 5 hours of sleep on nights when she has to work the next day, and 6-7 hours of sleep on nights when she does not have work. She does not feel fully rested in the morning, regardless of how much sleep she gets. She is a college graduate with no history of attention deficit hyperactivity disorder (ADHD), learning disabilities, drug or alcohol abuse, or mental health concerns. She spent 7 years working at airfields with substantial noise exposure, and had 1 concussion during combatives training from which she says she made a full recovery. She reports no family history of early onset dementia or other neurological conditions.

On exam, she appears physically fit and euthymic, though fatigued. Her bedside cognitive performance testing is normal (MOCA 29/30), as are her cranial nerves, visual fields, motor, sensory, and reflex testing. Her visual acuity is measured at 20/25 in each eye; she does not use corrective lenses.

Assessments:

1. “Relative” attention deficit, meaning that although she may not have a diagnosis of attention deficit disorder as defined in a medical context (i.e. she does not have a “disease”), her capacity for sustained attention is not adequate to meet the requirements of her situation.
2. Insomnia secondary to situational anxiety and caffeine misuse. Sleeping 5-7 hours per night is common but not optimal for cognitive performance. Her worries about workplace performance are also common for people with high-demand, high-consequence careers and do not necessarily indicate an anxiety disorder, but they are likely to be interfering with sleep quality. Excessive caffeine use (>400 mg /day) and caffeine use late in the day (3 pm) are also highly likely to be counterproductive in that the impairment of sleep likely outweighs the benefits during the daytime.
3. Possible alcohol misuse. For optimal cognitive performance, most people should typically not drink more than 1 alcoholic drink in a 24-hour period. More alcohol than that can cause residual cognitive impairment and reduce sleep quality.
4. Suboptimal physical exercise: Cognitive performance in many domains is improved with 30-60 min of moderately intense cardiovascular exercise or strength training every day. Intermittent exercise (e.g. “weekend warrior”) is better than no exercise, but not as good as physical exercise every day.
5. Mild visual acuity deficits. While 20/25 vision is adequate for driving and most tasks, the eye strain caused by compensation for uncorrected mild visual acuity deficits can be fatiguing.
6. Episodic migraine without aura. Migraine can be both triggered by cognitive overexertion, and also result in functional cognitive impairment since cogniphobia (‘it hurts to think’) causes avoidance of cognitively challenging tasks.
7. Possible seasonal affective disorder, indicated by subjective worsening in darker months of the year and work in a facility with no natural light.

Next steps: additional evaluations and initial treatments:

1. Obtain workplace collateral history. Her self-report consistent with relative attention deficit should be verified with one or more unbiased individuals who have directly observed her cognitive performance relative to her situational requirements. Stimulant medications are a cornerstone of cognitive performance optimization in the domain of attention deficit, but there are some individuals who may make a less-than-fully transparent complaint of attention deficit-like symptoms in order to acquire stimulant medications for other reason. Thus, external verification is important to distinguish *bona fide* relative attention deficit from drug seeking. Collateral source information is the most appropriate approach to verification; neither bedside cognitive testing (over 5-10 minutes) nor even formal neuropsychological testing (over 3-4 hours) are fully adequate to reveal concerns that primarily manifest during 8-12 hours of intense cognitive activity.
2. Obtain outside collateral history: a family member, roommate, friend, or other person may provide additional information related to attention deficit in other settings, alcohol and drug use, sleep disorders, or behavioral patterns. This information can help guide cognitive performance optimization. Suboptimal cognitive performance outside of work in other domains such as driving, shopping, and interpersonal interactions may point to a need for more aggressive intervention than had otherwise been considered.
3. Polysomnography. In addition to insomnia, she may also have another undiagnosed treatable sleep disorder. Restless leg syndrome, obstructive sleep apnea, and hyperarousal associated with nightmares are all common, can all be diagnosed objectively, and can all be at least partially treated. Insufficient sleep is one of the most common underlying contributors to suboptimal cognitive performance and we obtain polysomnography in nearly everyone interested in cognitive performance optimization at some point in their care.
4. Blood tests for thyroid function, vitamin B12 levels, comprehensive metabolic panel and complete blood count, if these have not been performed by her other care providers. A medical diagnosis is unlikely in this generally healthy young adult, but subclinical medical conditions can contribute to suboptimal cognitive performance, and can often be treated best when caught early.
5. Structured assessment for depression. This can be done via self-report tools (e.g. PHQ-9) or via formal diagnostic interview from psychologist, psychiatrist, social worker or other trained health care provider. Many individuals can be reluctant to initially bring up mood-related concerns, but untreated depression can impair cognitive performance, especially in the domain of mental fatigue.
6. Referral for corrective lenses. Many people can compensate for minor refractory errors, but compensation causes eye strain and increases mental fatigue.
7. Audiology evaluation. Hearing loss and tinnitus can increase cognitive demands. Both can be treated, at least in part, using hearing aids, which are increasingly commonly used by military service members who have had a history of substantial noise exposure. Even people with normal auditory thresholds on pure tone audiometry can benefit from low gain hearing aids in terms of ability to understand speech in noisy environments.

A note on evaluations that may not be necessary at this stage:

1. Brain imaging. In the absence of abnormalities on neurological exam or ‘red flag’ headache symptoms, it is very unlikely that brain MRI will reveal a treatable condition. In our experience, virtually every MRI scan ordered in this context will be normal, or if it reveals an abnormality, it is likely to be incidental and not related to the cognitive performance concerns. Incidental findings such as small pituitary adenomas, nonspecific periventricular white matter hyperintensities, minor vascular anomalies, and calcifications are more likely to cause anxiety and divert attention away from the main lines of assessment and treatment, and less likely to contribute beneficially to cognitive performance optimization. That being said, some individuals who are excessively anxious about their cognitive performance may be reassured by a normal brain MRI, so it is not wrong to order an MRI for purposes of “crossing every t.”
2. Formal neuropsychological testing. As noted above, formal neuropsychological testing may not reveal abnormalities in cases of relative attention deficit. It may be helpful when there are symptoms concerning for attention deficit *disorder* (i.e. trouble paying attention in many contexts, not just at the end of a long shift at work) or concern about other neurological conditions. Furthermore, when formal quantitative documentation of cognitive performance is required, in-person neuropsychological testing can be appropriate.
3. EEG: EEG is unlikely to reveal epileptiform abnormalities or other relevant findings in this case. Cognitive performance decrements due to subclinical seizures are more likely to be intermittent, rather than regular every day occurrences. They are also more likely to be sudden onset with gradual recovery afterwards, rather progressively worsening during long shifts. EEG is useful primarily when there are a specific clinical indicators of seizure disorder.
4. qEEG: There is no compelling evidence that quantitative EEG is effective for making a diagnosis or guiding specific treatment. qEEG-based biofeedback is commonly provided at some other facilities, but has not been found to be helpful in our experience.
5. Comprehensive endocrinological testing: In the absence of other physical signs and symptoms of endocrine dysfunction, assessment for hormone deficiencies other than hypothyroidism is very unlikely to uncover a treatable condition, and more likely to reveal an incidental finding that may cause anxiety and distract attention from more productive lines of assessment and treatment.

Results of the initial evaluations:

Her work supervisor provides collateral source information. The supervisor notes that she is generally productive but agrees that the quality of her work declines towards the end of the day. The supervisor also mentions that she did not adapt to the night shifts as well as some of the other officers, and that she may not be on track to be promoted as fast as would be considered optimal for her chosen career path.

Her partner with whom she lives notes that she may sometimes drink more than 3 drinks in a night on a special occasion, but reports no additional aberrant behaviors or cognitive concerns.

Her overnight polysomnography reveals sleep onset of 50 minutes, 1 awakening after 4 hours followed by return to sleep 45 minutes later, and a total sleep time of 5 hours despite opportunity to sleep for 8 hours. There were no signs of sleep apnea, restless legs, or REM-related arousals.

Blood tests are all within normal limits.

PHQ-9 self-reported assessment for depression symptoms reveals a total score of 7, indicative of subclinical depression symptoms mainly in the domains of pessimism about the future, anxiety, and fatigue.

Optometry evaluation confirms suboptimal visual acuity and glasses for reading are prescribed.

Audiometry evaluation reveals mild high frequency hearing loss, and low gain hearing aids without wireless features (SCIF-compliant) are prescribed.

Next steps: The results are discussed with the patient, and a multidisciplinary treatment plan initiated at the second visit:

1. Cognitive behavioral therapy for insomnia (CBT-I). This psychological treatment program involving 8-12 weekly sessions with a therapist either in person on online is the first line treatment for insomnia. It is safer than sedative-hypnotic medications for chronic insomnia lasting more than a few days. Most sedative-hypnotic agents cause some cognitive impairment. Sedating antihistamines like diphenhydramine (Benadryl) in many over-the-counter sleeping pills have anti-cholinergic side effects. Benzodiazepines (Xanax etc.) can cause residual daytime sedation. Non-benzodiazepine “Z-drugs” (e.g. Ambien, Lunesta) can also cause daytime sleepiness, aberrant behavior, and other cognitive side effects.
2. Prescription for sustained release melatonin 2 mg each night for insomnia. This has a small but measurable benefit on sleep quality and cognitive performance in many individuals. Unlike sedative-hypnotic agents, it is safe for long-term use. In our experience, it can be a useful adjunct to CBT-I, but not sufficient by itself to fully address insomnia.
3. A “prescription” for morning bright light in the form of recommendation to buy and install a 10,000-12,000-lumen full spectrum lamp of the type used to treat seasonal affective disorder. The lamp should be used for 30-45 min each morning, held about 18 inches away from the face at a 45-degree angle (it’s too bright to look right at it). This treatment improves arousal during the day, and sleep quality at night even in the absence of true seasonal affective disorder in settings when natural morning light is not available. This can be especially important for people who do not get enough natural daylight such as SCIF workers.
4. Motivational interviewing with regard to alcohol use. Questions like “how much alcohol do you think is optimal for cognitive performance?” and “do you typically feel sharper or not as sharp when you wake up in the morning after you’ve been drinking?” can help insightful people understand the effects of alcohol. This approach has been more effective, in our experience, than simple prescriptive advice to consume no more than 1 alcoholic drink per 24-hour period. We certainly share best available evidence when asked, but do so in a non-judgmental fashion.
5. Motivational interviewing with regard to exercise. Similarly, questions like “do you typically perform better or worse at work on days when you exercise?” and “what times during the work day can you get away for 5-10 minutes of exercise?” help build insight and encourage positive behavioral change. Pragmatic sensitivity is required. Suggesting going to the gym before or after work to a person who starts at 0430 and ends at 1630 is not likely to help. However, even small amounts of exercise can be beneficial; most supervisors will allow a 5-minute break every hour or so during a 12-hour day for a fast walk, a stair climbing session, resistance bands, or indoor calisthenics.
6. Discussion about optimal caffeine use. This is part of cognitive behavioral therapy for insomnia, but it is too important to wait for therapist’s availability. It logically goes well after discussions about melatonin, bright light, alcohol, and exercise, since people are reluctant to consider reducing caffeine use until they have a good alternative plan for managing fatigue. Optimal caffeine use typically involves no more than 400 mg per day total (e.g. 2 typical energy drinks), and no caffeine use within 8 hours of desired sleep time (e.g. none past 1 pm for someone who wants to be asleep by 9 pm). Since people become tolerant to caffeine quickly, it is ideal to take 1 whole day off of caffeine each week, or at least to reduce caffeine use to the minimum amount required to prevent caffeine withdrawal side effects like headaches and mood instability.
7. Encouragement to wear the prescription glasses and use the hearing aids. Explain the role of eye strain, hearing loss, and tinnitus in contributing to cognitive load.
8. Prescription of an oral triptan for migraine, with instructions to use it as soon as possible on onset of warning that a migraine is coming on. Triptans are generally safe in healthy young adults, and often more effective than ibuprofen for migraine headaches when used early. As noted above, migraines can impair functional cognitive performance even in the absence of neurological aura.

Third visit, follow-up:

After 6 weeks, she reports that she is partway through cognitive behavioral therapy for insomnia, taking the melatonin each night, using the bright light for 15-20 minutes each day, reducing alcohol use, taking breaks each day at work to do pushups or air squats for 5 minutes, wearing her new glasses, and using her hearing aids. She reports that her cognitive performance is a bit better, but still not where it needs to be. She has not been able to reduce her caffeine use. She has had 1 migraine headache, took the triptan, and had relief of symptoms such that she did not have to miss work.

At this point, the clinician has three main choices: The conservative approach would be to have her finish the cognitive behavioral therapy for insomnia in order to attempt to fully treat her insomnia before adding any new interventions. The aggressive approach would be to consider a adding a prescription stimulant such as methylphenidate at this visit. An intermediate approach would be to consider adding a more modest pharmacological intervention such as atomoxetine or modafinil at this visit. The clinician discusses the pro’s and con’s of these three paths forward with her over 15-30 minutes. She expresses as strong preference for the second path involving stimulants like methylphenidate, since her current workload includes tasks that have mission-critical national security importance. She has heard than many of her co-workers are using these medications. Furthermore, she notes that her co-workers are already fully loaded with their own tasks and cannot continue to assist her indefinitely.

In our experience, the first conversation about stimulants like methylphenidate usually includes the following components:

1. An overview: prescription stimulants like methylphenidate can have meaningful benefits, but they also have real risks and can create substantial inconveniences.
2. The risks, which are significant but usually manageable in otherwise healthy adults include:
   1. Increased blood pressure and heart rate. Often not safe for people with uncontrolled hypertension, uncontrolled coronary artery disease, or significant risk of cardiac arrhythmias. Many of the cardiovascular adverse effects of methylphenidate can be at least partially counterbalanced by immediate release metoprolol, which has similar pharmacokinetics as immediate release methylphenidate. Regular cardiovascular exercise is also an important countermeasure.
   2. Increased frequency and severity of headaches. Often not a good idea for people with poorly controlled headache disorders. Commonly, we work with patients interested in cognitive performance optimization to get headaches under good control first before starting methylphenidate.
   3. Increased anxiety, especially at peak dose. Prescription stimulants like methylphenidate can do more harm than good for patients who are over the top of the Yerkes-Dotson curve, i.e., their level of arousal is so high that more stimulation worsens their cognitive performance. In this situation, effective treatment for anxiety disorders is typically required before stimulants can be considered. However, there is recent data from a small randomized controlled trial, corroborated by our own experience, indicating that patients with post-traumatic stress disorder (PTSD) actually *benefit* from methylphenidate. This result was initially counterintuitive, but subsequently several of our patients have told us that they are able to concentrate better in PTSD therapy and got more out of their sessions with their behavioral health providers. Furthermore, some of the anxiety related to work performance can also improve with methylphenidate. Several of our patients have told us that they used to lie awake at night worrying about whether they were going to make mistakes at work the next day. With the cognitive performance benefits provided by methylphenidate, they report more self-confidence and less nighttime rumination.
   4. Decreased sleep. Insomnia is a major concern when prescription stimulants like methylphenidate are not used correctly. We typically prescribe immediate release methylphenidate in two daily doses; one in the morning and one near noon. Typically, the effects of immediate release methylphenidate are fully worn off in 4-6 hours, so when the last dose of the day is given no later than 8 hours before bedtime, sleep is not usually adversely affected. We also prescribe them to be taken 5-6 days per week, not 7. Patients who wish to nap in the afternoon on weekends or holidays should not take any methylphenidate or take only a single early morning dose on those days.
   5. Tremor. Typically, not impairing, but can be unpleasant. Can often be mitigated with low dose beta blockers.
   6. Decreased appetite. This can be a risk or a benefit, depending on the circumstances. We assess patients to make sure they do not have unwanted weight loss and are not becoming malnourished. This is uncommon in otherwise healthy adults.
   7. Risk of addiction. This is very low in our experience for patients using methylphenidate for legitimate cognitive performance optimization. It is reduced further by using the medications 5-6 days per week, not 7, and taking at least 1-2 full weeks off each year.
   8. Risk of theft. Prescription stimulants, especially Adderall, have a street value and are often stolen. We advise patients to minimize the number of people who know they have prescriptions, store the pill bottles securely at home, and only carry 1 extra pill to work if needed for a noon-time dose.
   9. Very small increased risk of seizures. We typically do not use prescription stimulants like methylphenidate for cognitive performance optimization in patients with seizure disorders or with brain lesions that put them at high risk of seizures.
   10. Very small increased risk of psychosis. Likewise, we typically do not use prescription stimulants like methylphenidate for cognitive performance optimization in patients with psychotic disorders. However, we have had patients with PTSD-related hallucinations whose hallucinations worsened slightly with methylphenidate, but the risk/benefit ratio was still favorable since the PTSD-related hallucinations were still manageable.
3. The inconveniences, which are real but usually acceptable if the benefits are worthwhile:
   1. We cannot provide refills of prescription stimulants like methylphenidate. We need to see the patients frequently during the dose adjustment, and every 3 months when they are on steady state dosing.
   2. Overseas deployment for Military Service Members requires a waiver. This means that Service Members will need to have a detailed discussion about the risks and benefits with their commanding officers.
   3. Not all pharmacies carry prescription stimulants. There are frequent shortages, and patients often need to call around to multiple pharmacies.
   4. Prescription stimulants will appear in the urine during drug testing. Patients need to disclose that they are taking prescription stimulants when asked in the context of a security clearance or other drug testing.
   5. Prescription stimulants can be subject to travel restrictions. Patients need to carry the medications in their original bottles during travel. Some medications that are legal in the US are not legal in other countries (e.g., mixed amphetamine salts or brand name Adderall are not legal in Japan).
4. The most common benefits, based both on published literature in other contexts and on our experience:
   1. Increased sustained focus
   2. Greater attention to detail
   3. Improved ability to get back on task after interruptions or distractions
   4. Faster information processing
   5. Reduced mental and physical fatigue
   6. Improved confidence in one’s cognitive abilities
   7. Anti-depressant effects, especially when suboptimal workplace performance is a major stressor.
   8. Increased motivation to perform regular physical exercise, which is also an important contributor to cognitive performance optimization.
5. Dosing: start low and increase carefully.
   1. We typically start with 5 mg of immediate release methylphenidate each morning and 5 mg each day near noon. This is a test dose that doesn’t typically have any meaningful benefits. We use it to make sure that patients are not hypersensitive or allergic to methylphenidate.
   2. We prescribe stimulants for use 5-6 days per week, not 7. There should almost always be at least one whole day off each week unless there are unusual circumstances. We tell patients and their families not to have too many expectations for the day off.
   3. Check for side effects in person, with additional collateral history. This usually takes only 5 minutes, but it is important for safety.
   4. To titrate up fast, we increase by 5 mg per dose every 3-7 days. The maximum dose is usually 0.3 mg/kg, so around 15 mg for a 130-pound person, 20 mg for a 160-pound person, 25 mg for a 180-pound person, and 30 mg for a 210-pound person. We titrate up fast when the mission-critical nature of the cognitive tasks requires optimal benefit as soon as possible, without compromising safety. This can be inconvenient since we need to perform at least a brief physical exam before each dose increase.
   5. To titrate more slowly, we increase every 4 weeks. This is often the most common pace for patients who can’t come back to clinic more frequently.
6. Options
   1. Switch to a long-acting methylphenidate formulation. Some individual who have a strong peak effect followed by substantial fatigue as the drug wears off can benefit from sustained release formulations of methylphenidate at the same total dose. Interference with sleep is the main limitation of this approach since the duration of action for sustained release formulations can be variable. We also commonly prescribe an intermediate-long acting formulation for the morning dose, and then a short acting formulation in the afternoon. There are many options for longer-acting formulations including pills, liquid, and transdermal patch forms.
   2. Optional trial of mixed amphetamine salts (Adderall). Typically, methylphenidate and mixed amphetamine salts are similarly effective in our experience. Occasionally the mixed amphetamine salts are more effective, though they may also be more problematic due to higher risk of theft, pharmacy shortages, and legal issues in some countries.
   3. Optional trial of lisdexamfetamine (Vyvanse). Again, typically, methylphenidate and lisdexamfetamine are similarly effective in our experience, but occasionally lisdexamphetamine is more effective.

She asks several appropriate questions, discusses the information with her partner by phone, and requests a trial of methylphenidate. She is prescribed 5 mg methylphenidate each morning at 0430 and 5 mg each day at 1100. She is instructed to continue with cognitive behavioral therapy for insomnia, daily exercise, melatonin, morning bright light, glasses and hearing aids. She is instructed to try cut down her caffeine use later in the day. She is motivated to improve as fast as possible, and is willing to come back to clinic each week for brief visits.

Visit 4: She returns to clinic and reports than the 5 mg methylphenidate has had no adverse effects, but no benefits either. Her neurological exam is unchanged. Her blood pressure and heart rate are unchanged 90 minutes after taking her morning dose. She is prescribed 10 mg methylphenidate each morning at 0430 and 10 mg each day at 1100, with instructions to take the weekend off and follow-up again in 1 week.

Visit 5: She reports that she notices some benefit of the methylphenidate in the morning, with effects starting about 30 min after the dose, and lasting until noon. She notices less benefit from the afternoon dose. She reports no concerns when asked about each of the above noted adverse effects in detail. Her blood pressure and heart rate are again unchanged 90 minutes after taking her morning dose. Her neurological exam is unchanged overall, though she appears less fatigued. She still has not been able to reduce caffeine use. She is prescribed 15 mg methylphenidate each morning at 0430 and 15 mg each day at 1100, with instructions to again take the weekend off and follow-up again in 1 week.

Visit 6: She reports substantial benefits both in the morning and afternoon in the domains of attention to detail, sustained focus, and mood. She still reports being fatigued by 3 pm, and cannot accomplish much during the last hour of her shift at work. She reports no adverse effects. She reports that she has reduced her caffeine use to half an energy drink three times a day. She reports that her sleep is improving, which she attributes to the combination of less caffeine, more exercise, and less anxiety. Her systolic blood pressure is increased by approximately 5 mM Hg and her heart rate is increased by approximately 5 beats per minutes 90 minutes after taking her morning dose. Her neurological exam is notable for faster responses overall, with no tremor or other abnormalities. She is satisfied with this dose and does not wish to increase further, even though by weight-based dosing she could increase to 20 mg twice a day.

Subsequent visits every 3 months: She maintains a stable dose of methylphenidate at 15 mg twice a day 5 days per week, with no methylphenidate on weekends. She takes 1 whole week off during vacation and notices that she feels less motivated than during the work week. Follow-up collateral history from her supervisor indicates that her work performance has improved and she has not made any serious errors. She has been promoted and given more responsibility at work, but feels confident that she can handle the increased load. She reports migraine headaches once or twice per month, especially related to intense cognitive activity. These respond to sumatriptan. She reports no other adverse effects. She completes cognitive behavioral therapy for insomnia and finds it beneficial. She reduces her caffeine use to half an energy drink twice a day with none past 12 noon, which further improves her sleep. Her neurological exam and vital signs remain unchanged. She continues daily exercise, melatonin, morning bright light, glasses and hearing aids.

After 1 year, she asks how long she will need to stay on methylphenidate. In order to help answer the question, she is instructed to take 1 whole work week off from methylphenidate but maintain her sleep, caffeine, exercise, and other activities exactly the same as usual during a week when there are no especially important activities scheduled. She does this for three days, and reports that her cognitive performance is substantially worse than while on methylphenidate, but better than before starting her multidisciplinary treatment plan. She reports that she restarted methylphenidate on the 4^th^ day because she had unexpected mission-critical tasks to perform and was afraid of making a mistake. She continues successfully on a stable treatment plan for several years.

Example 2: “Senior Leader”

A 59-year-old senior officer at the Pentagon presents with questions about whether he has “early onset Alzheimer’s.” His main concerns are that he has trouble remembering peoples’ names, keeping track of details, and organizing his priorities at work. He has been able to get his daily work done, but has not been able to execute larger more complex projects effectively. He also notes that he has become more short-tempered, both at work and at home. He has tried brain training exercises but did not find them helpful. He reports that he has recently been promoted to a position requiring much greater responsibility than he has had in the past, including overseas travel as well as managing a large and fractious staff. He denies issues with long term memory, reading, writing, mathematical abilities, or visuospatial skills, and has not made any serious mistakes. On further discussion, he recognizes that he has always had trouble recognizing and understanding other peoples’ emotions, but this has not been as much of a concern until he moved up to his current position.

His past medical history is notable for hypertension, hypercholesterolemia, and episodic migraine with typical visual aura. He takes lisinopril, atorvastatin, and rimegepant. He has a family history of dementia; his mother died with Alzheimer’s in her 80’s, and his father had a decline in memory starting in his late 60’s which was attributed to cerebrovascular disease. He drinks 1 glass of wine with dinner each evening and has 1 whiskey before bed each night. He falls asleep quickly at 2230 and wakes up with his alarm clock at 0500. He reports feeling tired on awakening. He exercises for 45 minutes every morning in his home gym before work. He drinks 4-5 cups of coffee throughout the day.

On exam, he appears large and muscular, euthymic, well groomed, and well dressed. His speech and language are normal. He scores a 30/30 on the MOCA. His visual acuity is 20/20 with glasses. Cranial nerves, visual fields, motor, sensory, reflexes, gait, and balance are all normal. His vital signs are within normal limits. He has a waist-to-hip ratio of 1.2 and his BMI is 31.5. His neck circumference is 17 inches.

He has had an MRI scan of the brain ordered by his primary care provider which shows mild generalized atrophy and 5 small foci of elevated T2/FLAIR signal in the deep white matter, interpreted as nonspecific and normal for his age. He has had recent labs including CBC, CMP, TSH, T4, Vitamin B12, testosterone, and PSA which were all normal.

His wife was reached by phone to provide collateral information. She notes that he has always been somewhat irritable with her, but that it has gotten worse recently and has been losing track of scheduled events more than in the past. She thinks he sometimes has 2 or 3 whiskeys before bed. She notes that he snores, but she does not think he stops breathing in his sleep. She reports that his concentration is worse when he has had a headache earlier in the day, even if he says that his head doesn’t hurt anymore. This occurs 1-2 times per week. When asked about this, he agrees that his cognitive function is not as good on headache days. She also notes that he has been trying to lose weight by diet and exercise for several years, but has continued to gain about 1-2 pounds per year since he was in his 40’s. She reports that he is about 35 pounds heavier than when they were first married.

Initial assessment:

1. Possible mild cognitive impairment, especially in the domains of executive function and emotional intelligence.
2. Possible obstructive sleep apnea, based on body habitus and collateral source report of snoring.
3. Obesity, according to BMI-based definition and waist-to-hip ratio.
4. Episodic migraine with possible cognitive impairment during postdrome.
5. Possible early cerebrovascular disease.

Initial Plan

1. Referral for formal neuropsychological testing due to the concern for early onset Alzheimer’s disease, with assessment of executive function and theory of mind.
2. Referral for overnight polysomnography to evaluate for sleep apnea.
3. Referral for leadership coaching, with focus on emotional intelligence.
4. Referral to headache clinic for consideration of additional treatments to reduce frequency of migraines with cognitive impairment.
5. Motivational interviewing with regard to alcohol use.

Visit 2. He returns to clinic 6 weeks later with the following results:

1. Neuropsychological testing reveals performance within normal ranges in all domains. The testing shows relative strengths in visuospatial and linguistic skills, and slightly below average performance in theory-of-mind tests.
2. Polysomnography reveals moderately severe obstructive sleep apnea. The sleep medicine clinic recommends weight loss and provides a prescription for CPAP. He received a CPAP machine but finds it annoying and feels that he is not getting a good night sleep when wearing it.
3. Leadership coaching engages him in a series of role-playing exercises focusing on emotional intelligence. He finds it very useful, and begins incorporating the new skills into his daily routine.
4. The headache specialist obtains additional detailed history and makes the diagnosis of migraine with aura involving cognitive impairment. After discussion of alternatives, risks and benefits, the headache specialist recommends botulinum toxin injections into the head, neck, and shoulders for migraine prophylaxis. It is explained that generally, people with less than 3 migraine headaches per week do not need prophylaxis. However, under some circumstances, prophylaxis can be appropriate even when migraines are infrequent. In his case, for example, as a mission-critical decision maker, he may benefit from prophylaxis when the consequences of cognitively-impairing migraines could have national security implications. This is especially relevant for migraine with neurological aura since abortive medications are more effective for headache pain than for aura.
5. He has thought carefully about his alcohol use, discussed it with his wife, and decides to stop drinking for 1 month as an experiment.

Overall, he feels confident that he is on the right track, but has not yet noticed substantial improvements in his cognitive performance.

Plans:

1. Encouragement to continue his trial of CPAP for sleep apnea, with follow-ups at the sleep medicine clinic to adjust settings.
2. Discussion of a prescription for tirzepatide for weight loss. This GLP-1 agonist was recently approved by the FDA for moderate to severe sleep apnea in adults with obesity. He is counseled about the most common side effects including nausea, diarrhea, vomiting, constipation, abdominal pain, indigestion, injection site reactions, feeling tired, allergic reactions, belching, hair loss, and heartburn. He agrees and is prescribed 2.5 mg per week, increasing by 2.5 mg per week every 4 weeks up to 10 mg per week. Tricare prior authorization is requested and approved based on his BMI, sleep apnea diagnosis, contraindications to other weight loss medications (history of hypertension), and attempts to make lifestyle changes for more than 6 months.
3. Continue leadership coaching
4. Botulinum toxin injections for migraine prophylaxis.
5. Continue to abstain from alcohol, or have at most 1 drink per 24-hour period.

Visit 3: He returns to clinic 8 weeks later reporting substantial improvements. He has been performing better at work and is beginning tackle major long-term mission-critical initiatives. His wife confirms that he is less irritable at home as well. He reports that he has lost 11 pounds and is sleeping better. He has decided not to use the CPAP, and instead concentrate on weight loss. He has not had any serious side effects from the tirzepatide. He has finished 6 leadership coaching sessions and now feels he can incorporate the strategies he has learned himself on his own, with occasional brief check-ins with his coach and his own chain of command. He reports that he has had only 2 headaches in the 4 weeks since receiving the botulinum toxin injections (reduced from 1-2 per week) and has another set of injections scheduled for 12 weeks after the first set. He did not have any adverse reactions to the botulinum toxin injections. He has not been drinking alcohol; he says that he misses it, but recognizes that it is not good for his cognitive performance at his age.

He brings up a new concern. Over the next 6 months, he will need to do a substantial amount of overseas travel, including meetings with allies in Asia. Due to Dept of Defense travel regulations, he is not allowed to book business class tickets, and is afraid that he will be very fatigued on arrival. He reports that he often doesn’t sleep well in unfamiliar places, and has never been good at adapting to jet lag.

Plan:

1. Continue tirzepatide 10 mg per week
2. Continue botulinum toxin injections
3. Continue to abstain from alcohol.
4. Extensive discussion about cognitive performance optimization during overseas travel, including the following elements:
   1. Recommendation to ‘sleep bank’ prior to a trip with expected sleep deprivation. Sleep banking can be done in many ways, but one recommendation is to sleep 1-2 hours extra each night for 1 week prior to the period of expected sleep deprivation.
   2. Recommendation to practice meditation and relaxation exercises at bedtime at home, in preparation for using these techniques to help with sleep in unfamiliar environments.
   3. Recommendation to purchase a small portable full spectrum bright light box to use to help with circadian phase shift.
   4. Recommendation to break up travel into segments (e.g. stopover in Hawaii on the way to Asia) and build in 1 rest day on arrival.
   5. Recommendation for early morning exercise on arrival in a new location to help with circadian phase shift. Recommendation to book and pay for a session with a personal trainer to exercise outdoors as a method to increase accountability; if he has already paid for the session he is more likely to actually exercise even if he doesn’t feel like it.
   6. Prescription for melatonin 2 mg sustained release at bedtime as needed for insomnia in the setting of jet lag.
   7. Prescription for suvorexant 10-20 mg at bedtime as needed for insomnia in the setting of jet lag with circadian phase shift of 6 hours or more.
   8. Prescription for modafinil 200 mg on awakening in the setting of jet lag after circadian phase shift of 6 hours or more to improve wakefulness.

Visit 4: He returns to clinic 10 weeks later and reports continued improvements. His long-term projects at the Pentagon are going well overall. He has lost another 9 pounds on tirzepatide. He has lost interest in alcohol, and has heard that others taking GLP-1 agonists have had the same effect. He is sleeping well at home, feels more refreshed each morning, and has reduced his coffee to 2-3 cups per day. He had recurrence of his typical 1-2 migraines per week about 10 weeks after his previous set of botulinum toxin injections, and had another set of injections with good results. His travel schedule has been intense, with 3 overseas trips in the past 10 weeks. He reports that the sleep banking before travel did not prove feasible. The meditation and relaxation have been modestly helpful for sleep. He was able to exercise on arrival for 2 of the 3 trips, and performed better when he did so. The melatonin had no effect whereas the suvorexant was modestly helpful. The modafinil helped him stay awake but did not improve his concentration when he was jetlagged. He stated that he did not want to try anything new, but wanted to continue on his current plan for another 3 months.

Visit 5: He returns to clinic 3 months later. Overall his cognitive performance has been stable. On the advice of a colleague, he tried using transcranial direct current stimulation but found that it had no effects. He has continued to lose weight. He continues to sleep well and is getting better at managing jet lag. His migraine headaches have been well controlled. However, his responsibilities at work have increased even further. He has now been asked to assist with leadership tasks outside of his area of expertise, stepping in to cover for other senior leaders who are on leave or assist others who are struggling. He finds the challenges involving learning about new projects, working with new teams, and facing new problems to be intellectually exciting, but cognitively fatiguing. He feels a strong sense of responsibility and is anxious about making a serious mistake with national security implications.

There is an extensive discussion with him, his wife, and his primary care provider about the possibility of using prescription stimulants such as methylphenidate for further cognitive performance optimization. The major concern in a 59-year-old with hypertension and hypercholesterolemia would be worsening of possible cardiovascular and cerebrovascular disease due to the increased heart rate and blood pressure effects. He is referred for a cardiac exercise stress test for cardiac risk stratification, plus brain and carotid magnetic resonance angiography for cerebrovascular risk stratification. It is agreed that if he has evidence of coronary artery disease or cerebrovascular disease, that he should address these risks before taking any prescription stimulants. If he does not have evidence of coronary artery disease or cerebrovascular disease, he may be offered a trial of low dose methylphenidate, including the option to counter-balance any heart rate and blood pressure effects using metoprolol or another peripherally acting selective beta blocker.

Visit 6: His cardiac exercise stress test reveals no evidence of ischemia. His brain and carotid magnetic resonance angiography are within normal limits. However, after a detailed discussion, he elects not to try the methylphenidate and instead focus on sleep optimization. He understands that he may re-consider methylphenidate in the future, but that it is not necessary for everyone.

1-year follow-up: At this point, he reports that he is doing very well and finally feels like he’s caught up on sleep after approximately 40 years of sleep deprivation. He has tried stopping tirzepatide, but he immediately gained weight and his sleep worsened so he restarted the medication. He continues to abstain from alcohol, exercise daily, and use his light box for travel-related circadian phase shifts. Overall, he feels that his cognitive performance is appropriate for his responsibilities.
